# Supplementary material for: Complexome profiling of the Chlamydomonas psb28 mutant reveals TEF5 as an early PSII assembly factor
Source: Plant Cell. 2025 Mar 17;37(6):koaf055. doi: 10.1093/plcell/koaf055 (PMC12164590; doi:10.1093/plcell/koaf055)
Supplement: koaf055_Supplementary_Data [file koaf055_supplementary_data.zip › psb28_interactive.zip!/plots/intensity/qualityPlots/peptides/FTSH1_Cre12.g485800.t1.2.html]

### Cre12.g485800.t1.2

Trivial name: FTSH1  
SeqCoverage [median]: 40.00  
Mass: 77.55 kDa  
  
 

| Sequence | MissedCleavage | Mass [Da] | wtIntensity | muIntensity | Score |
| --- | --- | --- | --- | --- | --- |
| AKDLVEQNIDILHK | 1 | 1634.90 | 54213.30 | 200477.10 | 220.85 |
| ALGKDVDLEK | 1 | 1086.59 | 86720.64 | 136437.73 | 118.77 |
| ATVVLPNDPDLVDILAK | 0 | 1792.00 | 43971.06 | 13135.65 | 163.09 |
| DAPGVSIPYQAA | 0 | 1187.58 | 3202.78 | 35235.46 | 128.28 |
| DGSQLQLTAVDGR | 0 | 1358.68 | 26342.55 | 89943.77 | 267.02 |
| DGSQLQLTAVDGRR | 1 | 1514.78 | 39799.17 | 97596.87 | 106.17 |
| DLVEQNIDILHK | 0 | 1435.77 | 23085.09 | 61294.10 | 115.14 |
| DVDLEK | 0 | 717.35 | 2367.67 | 4843.51 | 103.29 |
| EISKEEIADALER | 1 | 1501.76 | 59780.08 | 191262.18 | 136.24 |
| FSKDGSQLQLTAVDGR | 1 | 1720.87 | 2730.11 | 4918.56 | 82.66 |
| FSKDGSQLQLTAVDGRR | 2 | 1876.98 | 3313.70 | 18865.96 | 84.86 |
| GAAGGLTFFAPSEER | 0 | 1508.73 | 37178.34 | 89656.20 | 213.16 |
| GAAGGLTFFAPSEERLESGLYSR | 1 | 2414.19 | 20387.03 | 0.00 | 95.41 |
| GKALGKDVDLEK | 2 | 1271.71 | 6021.64 | 47898.77 | 152.38 |
| IAEELIFGEDDITTGASGDFQQVTR | 0 | 2711.29 | 10983.99 | 0.00 | 112.88 |
| IIAGPEK | 0 | 726.43 | 3919.58 | 14737.02 | 86.09 |
| IIAGPEKK | 1 | 854.52 | 15383.27 | 45689.50 | 111.82 |
| KDAPGVSIPYQAA | 1 | 1315.68 | 8431.48 | 33734.69 | 124.42 |
| LESGLYSR | 0 | 923.47 | 27503.12 | 46692.10 | 153.78 |
| LMVTQLGLSK | 0 | 1088.63 | 71521.75 | 173607.00 | 169.76 |
| LMVTQLGLSKK | 1 | 1216.72 | 13783.67 | 52106.98 | 155.99 |
| LVAYHEAGHALVGALMPEYDPVTK | 0 | 2580.30 | 15911.38 | 106047.35 | 112.36 |
| NLKEISKEEIADALER | 2 | 1856.98 | 22762.05 | 178231.75 | 136.01 |
| NPDKYTALGAK | 1 | 1176.61 | 5149.77 | 17405.90 | 69.72 |
| QVTVDRPDVQGR | 1 | 1368.71 | 168064.41 | 220265.86 | 106.01 |
| RATVVLPNDPDLVDILAK | 1 | 1948.10 | 26348.30 | 0.00 | 114.34 |
| RLVAYHEAGHALVGALMPEYDPVTK | 1 | 2736.41 | 0.00 | 6992.06 | 58.97 |
| RNLKEISKEEIADALER | 3 | 2013.09 | 0.00 | 5500.13 | 63.69 |
| RTPGFTGADLQNLMNEAAILAAR | 1 | 2429.25 | 25246.85 | 0.00 | 141.64 |
| SEYTLPEGNQWR | 0 | 1478.68 | 10961.27 | 50114.46 | 125.56 |
| SQNGGGGAGPMGGMGGAMDFGR | 0 | 1967.80 | 121.69 | 195.23 | 46.35 |
| TPGFTGADLQNLMNEAAILAAR | 0 | 2273.15 | 4875.55 | 0.00 | 128.74 |
| TYLENQMAVALGGR | 0 | 1521.76 | 79380.80 | 144631.24 | 331.48 |
| YSEFVNAVEAGK | 0 | 1312.63 | 6086.99 | 29836.05 | 185.08 |
| YSEFVNAVEAGKVER | 1 | 1696.84 | 85896.75 | 350177.43 | 172.43 |
|  |  |  | 1011445.9 | 2467530.6 |  |
